# Case Report: Definitive radiotherapy for inoperable anal Buschke–Löwenstein tumors: two cases with literature review

**DOI:** 10.3389/fonc.2026.1826399

**Published:** 2026-05-22

**Authors:** Mouna Ben Rejeb, Ghada Abdessatar, Ines Mlayah, Myriam Saadi, Nesrine Mejri, Lotfi Kochbati

**Affiliations:** 1 Radiation Oncology Department, Abderrahmen Mami Hospital, Ariana, Tunisia; 2 Faculty of Medicine of Tunis, University of Tunis El Manar, Tunis, Tunisia; 3 Medical Oncology Department, Abderrahmen Mami Hospital, Ariana, Tunisia

**Keywords:** Anal neoplasms, Buschke–Löwenstein tumor, case report, giant condyloma acuminatum, human papillomavirus, radiotherapy, volumetric modulated arc therapy

## Abstract

**Background:**

Buschke–Löwenstein tumors (BLTs), also known as giant condyloma acuminatum, are rare HPV-related lesions with benign histology but locally aggressive behavior, high recurrence rates, and risk of malignant transformation. Complete surgical excision is the standard treatment; however, surgery may be infeasible in advanced, recurrent, or anatomically complex cases. Evidence for radiotherapy as a primary treatment is limited.

**Case presentation:**

We report two patients with inoperable anal BLTs treated using modern volumetric-modulated arc therapy (VMAT). Patient 1 received concurrent chemoradiotherapy (30 Gy in 15 fractions) and achieved a marked clinical response with grade 2 acute radiomucositis. Patient 2 underwent definitive radiotherapy alone (45 Gy in 25 fractions) resulting in a partial clinical response at early follow-up, with minimal toxicity. Both treatments were well tolerated. We performed a literature review of published cases of BLTs treated with radiotherapy between 2000 and 2025, which demonstrated heterogeneous treatment approaches but generally favorable clinical responses and acceptable toxicity.

**Conclusions:**

Radiotherapy using contemporary VMAT techniques is feasible, safe, and effective for selected patients with inoperable BLTs. When surgery is not possible, radiotherapy alone or combined with chemotherapy may provide early clinical tumor regression with limited toxicity. These findings, combined with existing literature, support consideration of radiotherapy in multidisciplinary management of this rare condition.

## Introduction

The Buschke–Löwenstein tumor (BLT), also referred to as giant condyloma acuminatum, are rare, locally aggressive lesions arising from HPV-associated warty growths, most commonly HPV types 6 and 11 (1). Although histologically benign, BLTs can exhibit destructive local behavior, high recurrence rates, and, in 30–56% of cases, malignant transformation into squamous cell carcinoma (1, 2).

First described by Buschke and Löwenstein in 1925, these tumors occupy a gray zone between condyloma acuminatum and well-differentiated carcinoma (2–4). BLTs predominantly affect men and are more frequent in individuals with risk factors such as immunosuppression, diabetes, poor hygiene, smoking, or multiple sexual partners (1, 5).

Clinically, they present as large, exophytic, cauliflower-like masses in the anogenital region, posing a challenge for complete surgical excision, which is generally considered the standard treatment. When surgery is not feasible due to tumor extent, recurrence, or functional concerns, alternative therapeutic strategies are needed (3). Radiotherapy has historically been controversial because of concerns about malignant transformation, but recent reports suggest that it may be an effective option in selected cases.

Here, we report two cases of inoperable anal BLT treated with definitive radiotherapy using modern volumetric-modulated arc therapy (VMAT). This report aims to provide practical clinical insights regarding treatment feasibility, dose selection, and early clinical outcomes in patients who are not candidates for surgical management, while contextualizing these findings within the existing literature.

## Case 1

### Case description

**Figure 1:**
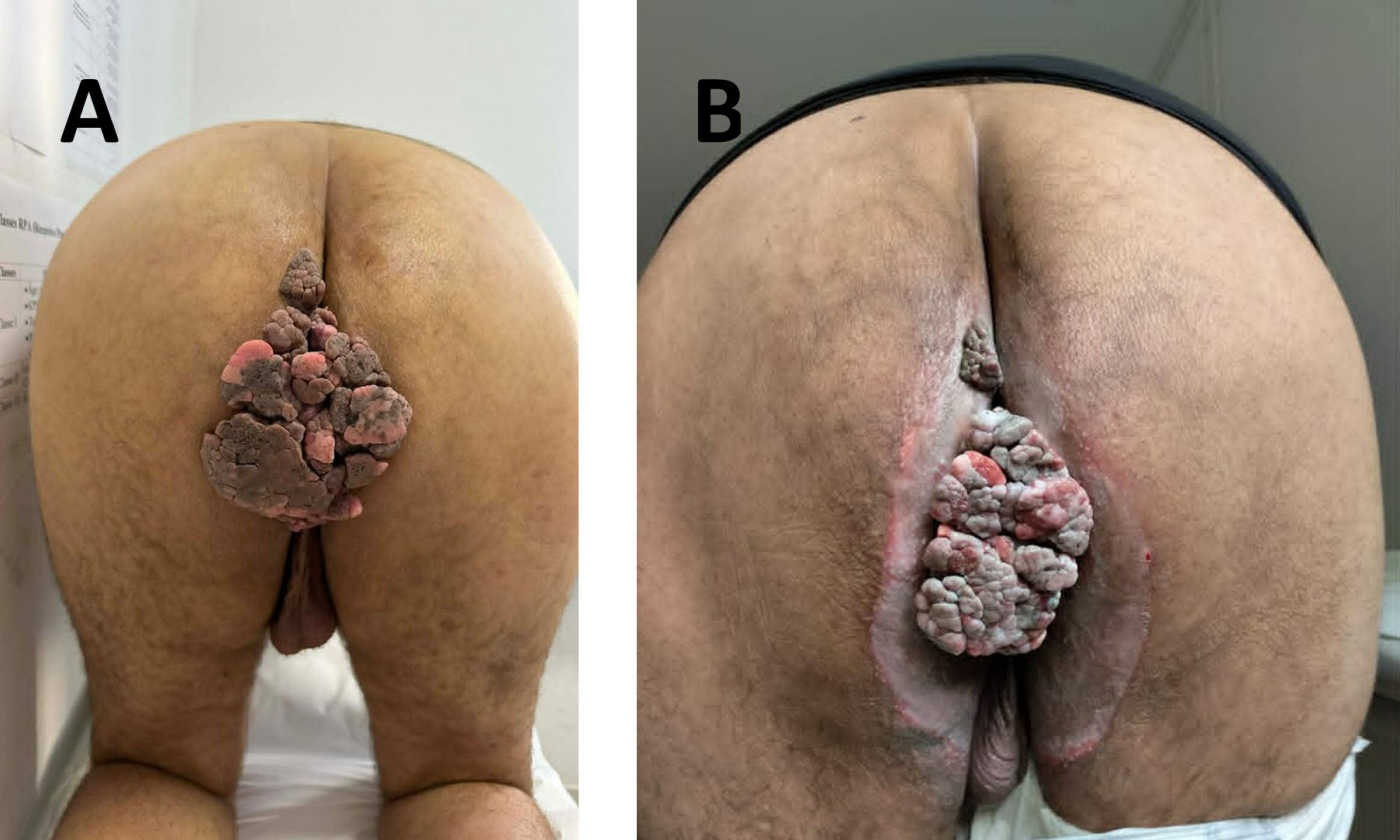
Clinical images of patient 1. **(A)** Recurrent large anal mass following incomplete surgical excision. **(B)** Partial clinical response of the recurrent anal mass at 3 months after exclusive VMAT radiotherapy.

A 68-year-old male with a medical history of type 2 diabetes managed with oral antidiabetic agents and coronary artery disease treated by stent placement presented with a progressively enlarging exophytic anal mass. The lesion measured approximately 8 cm and extended toward the base of the right scrotum (**Figure 1A**).

### Diagnostic assessment

A biopsy revealed epithelial proliferation with cytopathic features consistent with HPV infection, compatible with a markedly ulcerated condylomatous lesion, with no evidence of malignancy. Total colonoscopy showed an ulcerated anal margin lesion with a fistulous tract, with no additional abnormalities.

Pelvic MRI demonstrated an infiltrative soft tissue mass in the anterior perineal region, slightly left paramedian, measuring 63 × 22 × 46 mm, with intermediate T2 signal, low T1 signal, diffusion restriction, and contrast enhancement.

The lesion infiltrated the internal and external anal sphincters between the 12 and 1 o’clock positions (gynecologic position), involved the left puborectalis muscle, and contacted the left ischiopubic ramus without bone invasion. The lesion remained separate from the urethra, prostate, and penis.

Histopathological examination confirmed the diagnosis of Buschke–Löwenstein tumor, showing papillomatosis, acanthosis, and koilocytosis consistent with HPV infection, without evidence of stromal invasion. The main differential diagnoses included verrucous carcinoma and invasive squamous cell carcinoma, which were excluded based on histopathological findings.

### Therapeutic intervention

The case was discussed in a multidisciplinary tumor board, and the tumor was deemed inoperable. The patient received external beam radiotherapy delivered using volumetric-modulated arc therapy (VMAT). A total dose of 30 Gy was prescribed to the primary tumor, delivered in 15 fractions of 2 Gy, five fractions per week. No elective nodal irradiation was performed. Radiotherapy was administered concurrently with two cycles of chemotherapy consisting of 5-fluorouracil (800 mg/m²/day, continuous infusion on days 1–5) and cisplatin (100 mg/m² on day 2), repeated every 3 weeks. Treatment was well tolerated, with grade 2 acute radiomucositis as the only notable toxicity.

### Follow-up and outcomes

Pain intensity was assessed using a numeric rating scale (NRS) ranging from 0 (no pain) to 10 (worst imaginable pain), based on patient-reported symptoms documented during routine clinical evaluation.

Treatment was well tolerated except for grade 2 radiomucositis.

The patient reported severe pain before treatment, with an intensity of 9/10 on the Numeric Rating Scale (NRS). At 4 weeks after completion of radiotherapy, pain markedly improved to mild discomfort (NRS 2/10). Bleeding and difficulty with sitting also significantly decreased.

At the same follow-up time point, the patient demonstrated marked clinical regression of the visible anal mass on physical examination (**Figure 1B**). Response assessment was based on clinical evaluation, as post-treatment MRI was not performed at that time due to the early follow-up interval. These findings reflect early clinical improvement rather than definitive assessment of treatment response.

## Case 2

### Case description

**Figure 2:**
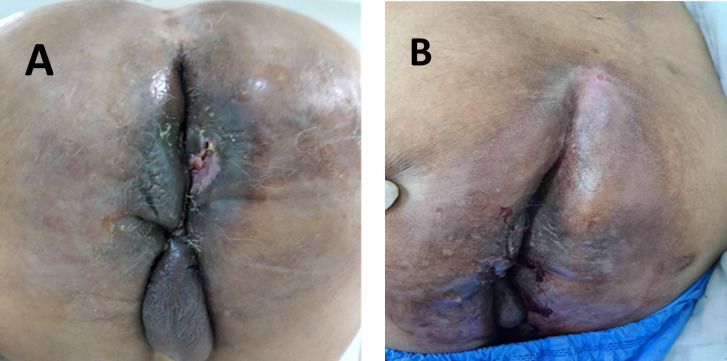
Clinical images of patient 2. **(A)** Exophytic 8 cm anal mass extending toward the base of the right scrotum at baseline. **(B)** Marked clinical regression of the anal mass at 4 weeks after VMAT radiotherapy with concurrent chemotherapy, associated with significant symptom improvement. Residual tissue changes remain visible at this early follow-up time point.

A 46-year-old male with a medical history of colonic diverticulosis had previously undergone complete surgical excision of a giant anal condyloma acuminatum. He later presented with a large recurrent anal mass following an incomplete surgical resection. The recurrence occurred approximately 3 months after the initial surgical procedure (**Figure 2A**).

### Diagnostic assessment

Pelvic MRI revealed a large perineal tumor centered on the anal margin, measuring approximately 117 × 106 mm in axial dimensions and extending 135 mm vertically.

The lesion showed heterogeneous enhancement, extended into the anal canal over 25 mm, and infiltrated both the internal and external sphincters at the distal third of the canal.

Risk factors and HIV status: The patient had no history of immunodeficiency. HIV serology was negative. There was no history of diabetes, immunosuppressive therapy, or smoking. The patient had a history of recurrent anal condyloma following prior incomplete surgical excision.

Histopathological examination confirmed the diagnosis of Buschke–Löwenstein tumor, showing papillomatosis, acanthosis, and koilocytosis consistent with HPV infection, without evidence of stromal invasion.

### Therapeutic intervention

The case was discussed in a multidisciplinary tumor board, which concluded that complete resection was not feasible.

The patient therefore underwent exclusive external beam radiotherapy delivered to the primary tumor using volumetric-modulated arc therapy (VMAT). A total dose of 45 Gy was prescribed, delivered in 25 fractions of 1.8 Gy, five fractions per week.

### Follow-up and outcomes

Treatment resulted in grade 2 radiomucositis.

The patient reported severe pain before treatment (NRS 8/10) associated with difficulty sitting. At 3 months post-treatment, pain decreased to mild intensity (NRS 2/10), with improvement in local symptoms, including reduced bleeding and better tolerance of sitting.

At this follow-up, a good partial clinical response of the anal mass was observed (**Figure 2B**). Post-treatment management will involve continued clinical surveillance with periodic reassessment in a multidisciplinary setting. The possibility of surgical management will be discussed depending on tumor evolution and resectability.

Response assessment was based on clinical evaluation, as post-treatment MRI was not performed at that time.

## Discussion

Buschke–Löwenstein tumors (BLTs), also known as giant condyloma acuminatum, are rare benign but locally aggressive lesions arising from pre-existing condylomatous warts associated predominantly with HPV types 6 and 11 (1). HPV infection remains the most common sexually transmitted infection worldwide, with prevalence increasing with multiple sexual partners and early sexual activity (2, 3). Most HPV infections are asymptomatic, with genital warts occurring in only 2% of cases, the risk is significantly higher in immunocompromised individuals (4). Reported risk factors for BLT include poor hygiene, immunosuppression (HIV infection or immunosuppressive therapies), chemotherapy, inflammatory bowel disease, perianal fistulas, smoking, diabetes, pregnancy, and low socioeconomic status (1, 5).

Macroscopically, BLTs present as large, exophytic, cauliflower-like masses in the anogenital region (anus, perineum, penis, scrotum, vulva, or vagina) with destructive growth that can mimic malignancy (6). Histologically, they are characterized by papillomatosis, marked acanthosis, hyperkeratosis, parakeratosis, and koilocytosis hallmarks of HPV infection. Importantly, unlike squamous cell carcinoma (SCC), BLTs demonstrate a preserved basement membrane and a lymphohistiocytic inflammatory infiltrate without evidence of stromal invasion (4). Therefore, although clinical examination and imaging with CT or MRI can strongly suggest the diagnosis, histopathological confirmation remains essential to differentiate BLT from SCC, which requires a markedly different therapeutic approach.

Despite being benign, BLTs are notorious for their high recurrence rates, even after complete excision. Radical surgical resection with tumor clear margins is widely regarded as the gold standard (4). A 2022 review by Purzycka-Bohdan et al., involving 42 cases, reported superior cure rates in patients treated with radical surgery compared with those managed with chemoradiotherapy with or without local excision (3). However, treatment selection must consider tumor size, depth of invasion, and the feasibility of achieving clear margins, particularly in recurrent or anatomically complex perianal lesions, such as in one of our patients where re-excision was not possible.

Nonsurgical modalities may be used in selected cases. Topical immunomodulators such as imiquimod have shown benefit in recurrent disease but carry local side effects, including erythema, burning, tenderness, and ulceration (7). Ablative techniques including cryotherapy and CO_2_ laser, are effective for small lesions and may be used as exclusive, neoadjuvant or adjuvant treatments (8). Neither of our patients was eligible for these modalities due to the extensive size of their tumors.

Systemic or intralesional therapies, including 5-FU, bleomycin, interferon, and cidofovir, have been reported in isolated case studies, mainly in immunocompromised patients. However, systemic toxicity remains a significant limitation, and evidence supporting their routine use is weak (3, 9).

Radiotherapy (RT) represents an alternative option in inoperable disease, as neoadjuvant treatment to reduce tumor burden, or as an exclusive modality, potentially combined with chemotherapy (1, 4, 10, 11). Existing data remain limited to case reports, and RT techniques vary widely, from cobalt-60 units to modern MV photon beams (12, 13).

## Role of radiotherapy in treatment strategy

Radiotherapy may play different roles in the management of Buschke–Löwenstein tumors depending on clinical presentation. It can be used as neoadjuvant therapy to reduce tumor size before surgery, as definitive treatment in patients who are not candidates for surgical resection, or as salvage therapy in cases of recurrence after prior surgery.

In the present report, radiotherapy was used as definitive treatment in both patients due to the inoperable nature of their tumors. These cases illustrate the potential role of modern radiotherapy techniques, such as VMAT, in providing local disease control and symptom control when surgical management is not feasible.

## Radiotherapy dose selection

Radiotherapy dose selection in Buschke–Löwenstein tumors remains highly variable across published reports, reflecting the absence of standardized treatment guidelines. Reported total doses range from approximately 30 Gy to more than 60 Gy, depending on tumor size, clinical context, and treatment intent (definitive, neoadjuvant, or palliative).

In our series, dose selection was individualized according to patient characteristics, tumor extent, and treatment objectives. A lower dose was used in Patient 1 due to comorbidities and the goal of symptom control, whereas a higher dose was delivered in Patient 2 to achieve sustained local tumor reduction in the setting of recurrent disease. These observations highlight the need for tailored treatment planning in this rare condition.

Compared with previously published cases, our report provides additional clinical observations supporting the feasibility and tolerability of radiotherapy delivered with contemporary techniques in patients with inoperable BLT. Notably, the radiation doses used were lower than those typically employed for anal carcinoma, reflecting the benign histology of BLT while still achieving clinically meaningful tumor regression. The dose of 30 Gy delivered with concurrent chemotherapy in Patient 1 was selected considering the patient’s comorbidities, locally advanced disease, and the goal of achieving symptom control while minimizing treatment-related toxicity. In the absence of standardized dose recommendations for BLT, treatment decisions were individualized based on clinical judgment and multidisciplinary team discussion. These findings suggest that radiotherapy, particularly when delivered using modern techniques such as VMAT, may represent a therapeutic option in carefully selected patients when surgery is not feasible. However, given the limited number of cases and short follow-up duration, these findings should be interpreted cautiously.

Between 2000 and 2025, 16 cases of Buschke–Löwenstein tumors treated with radiotherapy have been reported in the literature. Most were published as isolated case reports or very small series, with some cases associated with squamous cell carcinoma, as summarized in **Table 1**.

**Table 1:** Literature review of published cases of Buschke–Löwenstein tumors, with or without associated squamous cell carcinoma, treated with radiotherapy (2000–2025).

First author (year)	Sex/age	Tumor location	Pathology	Therapy	Radiotherapy details	Follow-up	Recurrence
Sobrado 2000 (13)	M/42	perianal	BLT	Definitive Radiation therapy	45 Gy in 25 fractions	20 months	No
Dolanc, 2002 (14)	F/56	perianal	BLT with SCC transformation	Radiotherapy, Abdominoperineal Resection	50 Gy in 25 fractions	NR	No
Chao, 2005 (15)	M/57	perianal	BLT with SCC transformation	Radiotherapy, 5FU, mitomycin	50.4Gy to primary tumor, 45Gy to perirectal LN, 36Gy to inguinal LN	1 year	No
Tytherleigh, 2006 (16)	M/40	perianal	BLT	Chemoradiation, Abdominoperineal Resection	NR	1 year	Yes
	M/51	perianal	BLT	Chemoradiation, Abdominoperineal Resection	NR	5 years	No
Handisurya, 2009 (17)	M/45	perianal	BLT with SCC transformation	Surgical intervention, Radiotherapy	60 Gy	6 months	Yes
Armstrong, 2009 (18)	M/46	perianal	BLT	Unsuccessful Abdominoperineal Resection, chemoradiotherapy	NR	34 months	No
Haque, 2009 (19)	M/38	perianal	BLT with focal transformation to verrucous carcinoma	Definitive Radiotherapy, 5FU, mitomycin	54Gy in 30 fractions	6 months	No
Indinnimeo, 2013 (11)	M/43	anorectal	BLT with SCC transformation	Radiotherapy, 5FU, mitomycin-C	45Gy (28 fractions) to the pelvis and a boost of 14.40Gy (1.8Gy/fr) to the primary tumor	3 years	No
	M/56	anorectal	BLT with SCC transformation	Radiotherapy, 5FU, mitomycin-C and local excision	45Gy (28 fractions) to the pelvis and a boost of 14.40Gy (1.8Gy/fr) to the primary tumor	1 year	No metastatic progression
	M/37	perianal	BLT with SCC transformation	Radiotherapy, 5FU, mitomycin-C and local excision	45Gy (28 fractions) to the pelvis and a boost of 14.40Gy (1.8Gy/fr) to the primary tumor	3 years	No
Shenoy, 2019 (20)	M/70	perianal	BLT with SCC transformation	Radiotherapy, 5FU, mitomycin	46Gy in 30 fractions	3 years	No
	M/58	perianal	BLT with SCC transformation	Radiotherapy, 5FU, mitomycin, diverting sigmoid colostomy	46Gy in 30 fractions	3 months	Yes disseminated disease death at 3 months
Chakrabarti, 2022 (12)	F/24	vulva	BLT	Definitive radiotherapy	60 Gy in 30 fractions	9 months	No
Sivapalan, 2024 (21)	M/39	anorectal	BLT	Definitive radiotherapy	50,4 Gy in 28 fractions	6 months	No
Wagle, 2025 (22)	M/46	anorectal	BLT	multiple surgical interventions, radiotherapy	50 Gy in 25 fractions	7 months	No

BLT, Buschke–Löwenstein tumor; M, male; F, female; SCC, squamous cell carcinoma; 5FU, fluorouracil.

Clinical experience with definitive radiotherapy in BLT suggests that treatment can induce rapid tumor regression and may provide local disease control, although long-term outcomes remain uncertain. Published reports indicate that clinically meaningful tumor shrinkage often occurs within weeks to a few months post-treatment (19, 22). Nevertheless, BLTs remain associated with high recurrence rates, reported up to 60%, and a risk of malignant transformation approaching 50%, particularly in HIV-positive patients. These findings underscore the necessity of long-term surveillance, including regular clinical and radiological assessments (1).

Preventive strategies, including HPV vaccination, early treatment of condylomatous lesions, sexual health education, and consistent condom use, are essential components of comprehensive management.

Limitations of this study include the retrospective nature of our case series, the small number of patients, and the short follow-up duration, particularly for Patient 1. The very short follow-up duration (4 weeks and 3 months) represents a major limitation, as it does not allow reliable assessment of response durability, recurrence risk, or long-term local control. Longer surveillance is required to better assess recurrence risk and treatment outcomes.

Another limitation is the absence of systematic post-treatment imaging, as response assessment relied primarily on clinical examination and symptom evolution. This may reduce the objectivity of response evaluation.

## Conclusion

Management of Buschke–Löwenstein tumors is challenging due to their rarity, aggressive local behavior, and high recurrence risk. While wide surgical excision with clear margins remains the preferred treatment, many patients are ineligible due to tumor size, invasion, or recurrence. Our experience suggests that radiotherapy, alone or combined with chemotherapy, may represent a feasible therapeutic option with acceptable toxicity in selected patients with inoperable BLT. However, conclusions regarding long-term disease control remain limited due to the short follow-up duration. Treatment should be individualized within a multidisciplinary team, with long-term follow-up given recurrence and malignant transformation risks. Further studies are needed to define optimal radiotherapy strategies and combination therapies for this rare condition.

## Patient perspective

Both patients reported good tolerance of treatment and noted improvement in symptoms following radiotherapy, particularly a reduction in pain, bleeding, local discomfort, and difficulty with sitting and defecation. Improvement in quality of life was assessed through clinical evaluation and patient-reported feedback during follow-up visits. No treatment interruption was required.

## Data Availability

The original contributions presented in the study are included in the article/supplementary material. Further inquiries can be directed to the corresponding author.
